# Emerging roles of circular RNAs in tuberculosis

**DOI:** 10.3389/fimmu.2022.995701

**Published:** 2022-09-20

**Authors:** Qinglan Wang, Danni Yang, Yinan Zuo, Dan Wang, Weimin Li

**Affiliations:** ^1^ Institute of Respiratory Health, Frontiers Science Center for Disease-related Molecular Network, West China Hospital, Sichuan University, Chengdu, China; ^2^ Department of Respiratory and Critical Care Medicine, Frontiers Science Center for Disease-related Molecular Network, West China Hospital, Sichuan University, Chengdu, China

**Keywords:** circular RNA, circRNA, tuberculosis, mycobacterium tuberculosis, immune response, biomarker

## Abstract

Tuberculosis (TB) remains a major global health issue, resulting in around 1.5 million people deaths each year. Better diagnostic and therapeutic tools are urgently needed. Circular RNAs (circRNAs) are a new class of noncoding RNAs with a covalently closed structure, and exhibit a tissue-, cell-, and developmental stage-specific expression pattern. Recently, circRNAs were thought to be regulatory molecules implicated in the onset and progression of a series of human diseases including tuberculosis. In tuberculosis, circRNAs have been shown to regulate host anti-TB immune responses, such as decreasing monocyte apoptosis, enhancing autophagy and promoting macrophage polarization. Importantly, circRNAs are physically stable and abundant in several types of body fluids. Therefore they are considered as promising minimally-invasive biomarkers. In this review, we focus on the recent advances in the immune regulatory roles of circRNAs, as well as their potential diagnostic value in TB.

## Introduction

Tuberculosis is the second leading infectious disease cause of death globally after COVID-19, caused by *Mycobacterium tuberculosis* (Mtb), which usually attacks the lung but can affect almost any part of the body. According to the WHO global TB report, approximately 10 million people fell ill with TB and 1.5 million people died from TB globally in 2020 (1). The COVID-19 pandemic largely impacts global TB control because of reduced access to care, leading to around 5% increase of TB deaths compared to 2018 (2).

Although the modern antibiotics and Bacillus Calmette–Guérin (BCG) vaccine dramatically help human beings to fight TB, it has still not been eradicated. The main reasons are that Mtb could develop drug resistance rapidly under the pressure of antibiotics and the BCG vaccine does not work well in adults (3, 4). Another reason is that Mtb is armed with a set of intricate immune escape mechanisms which enable the bacterium to avoid the host immune killing and to survive in host for a long time (5).

Circular RNAs (circRNAs) are a family of recently rediscovered RNA molecules. Originally thought of as mere byproducts of aberrant splicing (6), circRNAs are now appreciated to have important biological roles including regulating gene expression, modulating protein function, encoding proteins and so forth (7). While the roles of immune protein factors during Mtb infection have been extensively studied, the functions of circRNAs in TB remain relatively unclear. In this review, we provide an overview of the current understanding of circRNAs, with a particular focus on their functions in immune regulation. We introduce recent investigations that reveal new roles of host circRNAs in anti-TB immunity, and discuss the potential of circRNAs as novel TB diagnostic biomarkers.

## The discovery, biogenesis and function of circRNAs

### The discovery of CircRNAs

The first circRNA was discovered in 1976 on viroid particles (8). A few years later, circRNAs were observed by electron microscopy in the cytoplasm of eukaryotic cells (9). Nevertheless, they were mainly considered as “junk RNAs” generated from abnormal splicing events at that time (6). Until 1993, a testis-specific 1.3 kb circRNA from the sex-determining region Y (*Sry*) gene in mice was molecularly identified. This circRNA was considered to have a potential function in mouse testis (10). Further, next-generation sequencing (NGS) with specific protocols for library preparation ushered in the genome-wide profiling of circRNAs. It is now accepted that circRNAs are the predominant transcript isoforms from thousands of human genes rather than simply accidental byproducts of splicing (11), and their expression is conserved in eukaryotes (12).

### The biogenesis of CircRNAs

**Figure 1:**
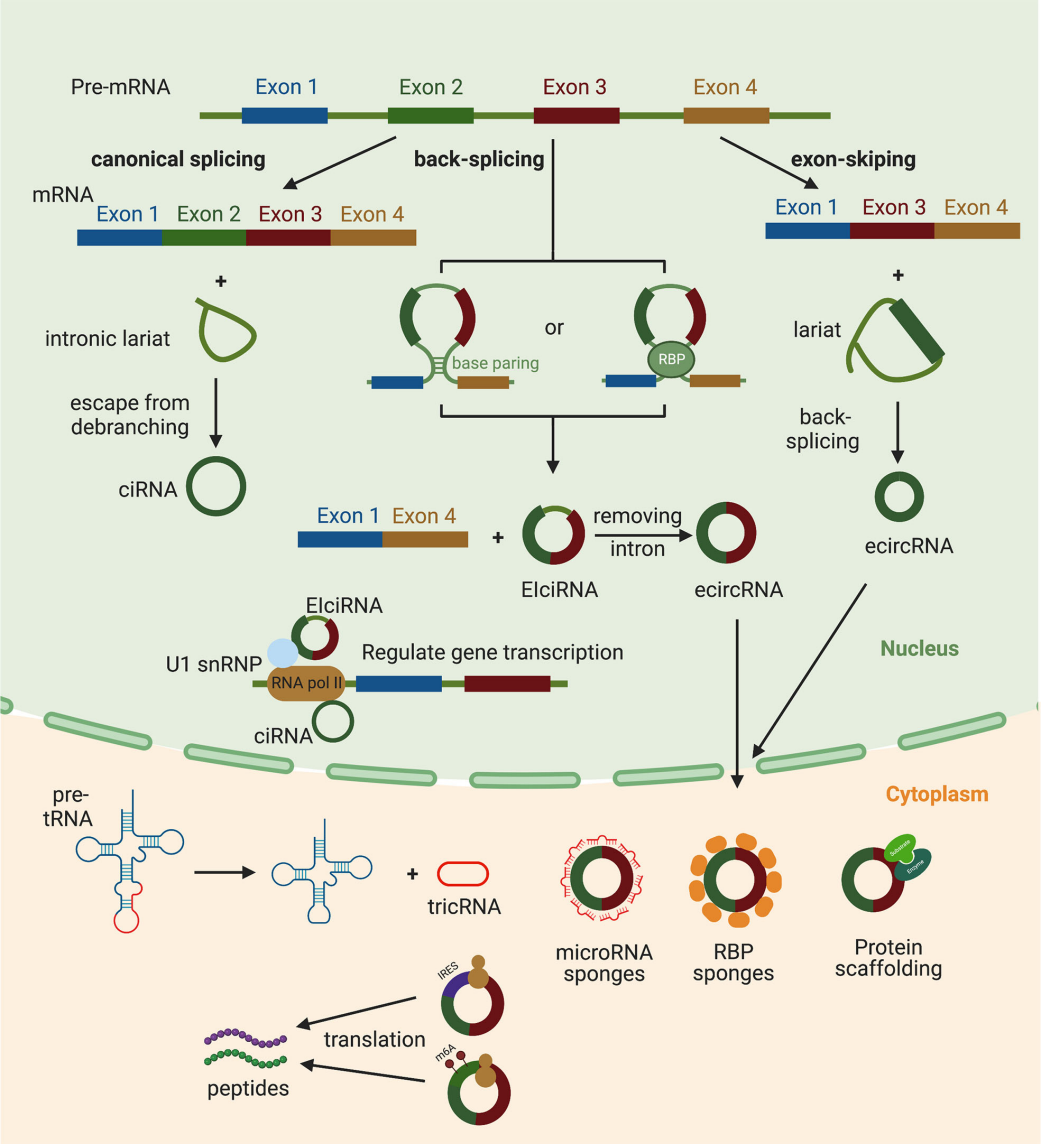
The biogenesis and functions of circRNAs. Exonic circRNAs (EcircRNAs) are generated by a non-canonical back-splicing which is favored by the base paring between reverse complementary sequences (such as Alu repeats) and the dimerization of RNA binding proteins (RBPs). EcircRNAs can also be produced from splicing intermediates called as lariat precursors that are created by an exon-skipping event. Circular intronic RNAs (ciRNAs) are generated from intronic lariats that escape from the debranching step of canonical linear splicing. tRNA intronic circular RNAs (tricRNAs) are formed during the process of pre-tRNA splicing. CiRNAs and EIciRNAs are located in nucleus. CiRNAs can interact with the RNA pol II complex and play a role in regulating parental gene transcription. EIciRNAs can interact with U1 small nuclear ribonucleoproteins and then increase the transcription of their host genes by binding with RNA pol II. EcircRNAs are translocated to cytoplasm after formation, and can act as microRNA sponges, RBP sponges, protein scaffoldings or templates of protein translation.

Based on their composition, circRNAs are currently divided into four categories: circular intronic RNAs (ciRNAs), exon–intron circRNAs (EIciRNAs), exonic circRNAs (ecircRNA) and tRNA intronic circular RNAs (tricRNAs) (**Figure 1**). The intronic lariat generated from canonical splicing is usually attacked by a debranching enzyme DBR1 and by exonucleases. Thus the cellular lariat is only an intermediate molecule that is usually rapidly degraded, but some lariats appear to be capable of evading this debranching process to form stable ciRNAs (13). ecircRNAs and EIciRNAs come from the back-splicing process wherein a downstream 5′ splice donor site is ligated to an upstream 3′ splice acceptor site (14), unlike canonical splicing that joins an upstream 5′ splice donor site with a downstream 3′ splice acceptor site. The circulation of pre-mRNA during back-splicing is mediated by the base paring between reverse complementary sequences of flanking introns or the dimerization of RNA binding proteins (RBPs) (15, 16). During the pre-mRNA transcription, exon skipping events may occur and forms an excised lariat. Subsequently, the lariat undergoes internal back-splicing leading to the formation of ecircRNAs or EIciRNAs if the flanking intronic sequences are retained in some circumstances (17, 18). EcircRNAs account for over 80% of the identified circRNAs and generally localize in the cytoplasm (9, 15, 19). In contrast, ciRNAs and EIciRNAs are usually localized in the nucleus (13, 18), suggesting that they may regulate gene transcription. TricRNAs are formed by tRNA introns through the cleavage of tRNA introns by tRNA splicing endonuclease (TSEN) complex and the circulation cleaved introns by an unknown ligase (20).

### The biological function of circRNAs

Although circRNAs were discovered decades ago, biological functions have only been studied for a small fraction of the circRNAs identified to date. Most of them were thought to act as miRNA sponges (19, 21–23) (**Figure 1**). Those circRNAs contain miRNA binding sites, which allow them to sponge miRNAs and restrain miRNAs’ function, thus indirectly regulating the translation of miRNAs’ target mRNAs. CiRS-7, which is highly expressed in the brain, contains more than 70 conserved binding sites for miR-7 (21). It has been reported to regulate the expression of miR-7 target genes and be involved in neuronal function. In addition, some circRNAs have also been demonstrated to act as RBPs’ sponges or protein scaffolds (24–27). The circMbl contains multiple conserved muscleblind (MBL) binding sites and thus is able to be specifically bound by MBL (28). The binding of MBL to circMbl decrease the available cellular MBL proteins levels. Its binding to the flanking introns of circMbl also facilitates the looping of mbl pre-mRNA and promotes the circMbl biogenesis which competes with the linear splicing of pre-mRNA, thus decreasing the production of mbl mRNA (28). By binding to both 3-phosphoinositide-dependent protein kinase 1 (PDK1) and its substrate AKT1, circ-Amotl1 can be as a protein scaffold to facilitate the PDK-1 dependent phosphorylation of AKT1 (25). Furthermore, circRNAs could also be translated into peptides or proteins in a cap-independent manner through internal ribosome entry sites (IRESs) (29–32). For example, circ-FBXW7 contains an ORF that can be translated into a novel 185-amino acids protein called FBXW-185aa, which has been reported to reduce the half-life of c-Myc and inhibit proliferation of cancer cells by interacting with the deubiquitinating enzyme USP28 (33). In addition to IRESs, m6A modification was also reported to be able to drive the translation of circRNAs (31).

## CircRNAs in immune regulation

As newly identified macromolecules, their roles in immune regulation attracted great attention. Some evidences supported that circRNAs are directly involved in immune regulation. Y. Grace Chen et al. first showed transfection of exogenous circRNA led to potent induction of innate immunity genes through the nucleic acid sensor RIG-I (34). Another study found that circRNAs can competitively bind and inhibit protein kinase R (PKR), a double-strand RNA activated enzyme in the antiviral signaling pathway, to regulate cellular immune signaling pathways extensively (35). Most cellular cicrRNAs have one or more intra-molecular imperfect RNA duplexes ranging from 16 to 26 bp, which is similar to double-strand RNA structures, to interact with PKR to suppress its activity. When cells are infected with viruses or stimulated by poly(I:C), RNase L degrades cellular circRNAs to release PKR, thus activating the downstream antiviral immunity mechanisms (35).

CircRNAs may also paly roles in the activation and function of immune cells. In response to different environmental stimuli, macrophages could be activated into pro-inflammatory M1-type macrophages or anti-inflammatory M2-type macrophages. One study investigated circRNAs expression profiles in M1- and M2-type macrophages using circRNA microarray. The authors identified 189 differentially expressed circRNAs, indicating that circRNAs may play important roles in regulating or maintaining macrophage polarization (36). Indeed, circRasGEF1B, an LPS-inducible cytoplasmic circRNA, has been reported to regulate the expression of intercellular adhesion molecule 1 (ICAM-1) in macrophages during LPS stimulation (37). ICAM-1 is known to recruit leukocytes to inflamed sites and mediate cell-to-cell interactions during antigen presentation (38, 39). In addition to macrophages, circRNAs’ expression profiles were also examined in neutrophils between healthy subjects and patients with asymptomatic Moyamoya disease (40). In this study, 123 circRNAs were identified differentially expressed between the two groups (40). Another comprehensive circRNA profiling study discovered that circRNA100783 is involved in chronic CD28-associated CD8^+^ T cell aging (41). It was also found that down-regulation of hsa_circ_0012919 increased the expression of DNMT1, decreased expression of CD70 and CD11a, in CD4^+^ T cells of patients with systemic lupus erythematous (42).

## The role of circRNAs in tuberculosis

Although excise roles of host circRNAs in TB remain to be further investigated, current evidences suggested that circRNAs could act as an important regulator in host anti-TB immune processes. Autophagy is an important cellular defense mechanism against intracellular pathogens including Mtb (43). Several circRNAs have been reported to induce autophagy in host cells. CircTRAPPC6B, one of the downregulated circRNAs in peripheral blood mononuclear cells (PBMCs) from active TB patients, could induce autophagy in Mtb‐infected macrophages by abolishing the repression of miR‐874‐3p on ATG16L1 expression (44). CircAGFG1, an upregulated circRNA in macrophages from TB patients, also has been reported to enhance autophagy whereas reduce apoptosis of Mtb-infected macrophages *via* the miRNA-1257/Notch axis (45). In contrast, hsa_circ_0045474, which is downregulated in monocytes from patients with TB, negatively regulates the autophagy level in macrophages likely by repressing the expression of miR-582-5p (46). Hsa_circRNA_103571 was also down-regulated in active TB patients. Bioinformatic analysis revealed that it has strong relationship with the biological process of autophagy by analyzing its potential target miRNAs and corresponding target genes of these miRNAs (47). CircRNA_101128 is highly expressed in PBMCs from active TB patients and negatively correlates with the level of its potential target miRNA let-7a, which is a known autophagy regulator, suggesting that it may play a role in regulating autophagy levels (48). Furthermore, some circRNAs, such as circ-Dnmt1, circCDYL, circMUC16, circPAN3, circ-0009910, circ0085131, circRACGAP1, circMOT1, cicr-0023404, circEIF6 and circ-0035483, are known to activate autophagy in cancer cells (49), but their roles in autophagy during Mtb infection remain unknown.

It has been known that macrophage polarization drives granuloma outcome during Mtb infection and Mtb has the potential to modulate macrophage polarization (50). An upregulated circRNA in TB patients, namely hsa_circ_0003528, was found to promote M1 to M2 macrophage polarization by sponging miR-324-5p, miR-224-5p and miR-488-5p and thus upregulating CTLA4 (51). Additionally, hsa-circRNA-100237 was indicated to play a role in TB pathogenesis by regulating macrophage activities (47). The potential mechanism is that the down-regulated hsa-circRNA-100237 in active TB patients could act as an miR-33 sponge, therefore promoting lipid storage by reducing mitochondrial fatty acid oxidation (47, 52). Circ_0001490 expression was down-regulated in the serum of TB patients and M.tb-infected THP-1 macrophages (53). Recently, it has been reported that circ_0001490 repressed M.tb survival and promoted the viability and inflammatory responses of THP-1 macrophages by regulating miR-579-3p/FSTL1 axis (53). CircPWWP2A, which is downregulated in Mtb-infected macrophages, was also reported to be able to protect human macrophages from Mtb-induced cytotoxicity by sponging miR-567 and thus abolishing the suppression of miR-567 on two pro-survival proteins, SIRT1 and PDK1 (54). Furthermore, the circRNA_051239 was reported to be significantly upregulated in drug-resistant TB patients (55). MiR-320a have three binding sites of circRNA_051239 and is significantly down-regulated in the drug-resistant patients (56). Thus, the circRNA_051239 may modulate the development of drug-resistance by targeting miR-320a.

The roles of circRNA in nontuberculous mycobacteria infection was also started to be investigated very recently. One study examined circRNA expression profiles of *Mycobacterium avium subsp. paratuberculosis* (MAP)-infected bovine monocyte-macrophages and uninfected cells by RNA sequencing (57). Authors identified 39 differentially expressed circRNAs between MAP-infected and uninfected macrophages, including 12 upregulated and 27 downregulated circRNAs in MAP-infected macrophages. Bioinformatic analysis showed that these cicrRNAs might play roles in Th1/Th2/Th17 cell differentiation, necroptosis, and JAK-STAT/chemokine signaling pathways. The other study screened circRNAs’ expression in osteocyte-like cells treated with N-glycosylated muramyl dipeptide (N.g MDP) from *Mycobacterium leprae* (*M. leprae*), trying to understand the mechanisms underlying the bone remodeling during *M. leprae* infection (58). In this study, 724 differentially expressed circRNAs and 724 differentially expressed messenger RNAs were identified, and 58 circRNA–miRNA–mRNA interaction pairs were obtained. The following analysis showed that these 58 genes are uniquely associated with “Circadian Rhythm” including *Clock*, which was known to be able to regulate bone formation, suggesting that circRNAs may paly roles in bone remodeling during *M. leprae* infection by regulating *Clock* genes’ expression.

Anti-tuberculosis drug-induced liver injury (ADLI) often leads to treatment interruptions. To explore ADLI-specific circRNAs, Biao Li *et al.* assessed the circRNA expression profiles in serums from TB patients with or without ADLI and in hepatocytes treated or untreated with anti-tuberculosis drugs (59). 113 co-differentially expressed circRNAs were identified in this screening. An upregulated circRNA among them, circMARS, was found to play roles in the compensatory repair mechanism of ADLI through the circMARS–miR-6808-5p/-6874-3p/-3157-5p–KMT2C–EGFR function axis. Another study published in this year found a down-regulated circRNA in ADLI patients called has_circ_0093884 could upregulate the expression of an anti-inflammatory protein SIRT1 by binding ribosomal protein S3 and thus regulate the hepatocyte inflammation in ADLI (60).

## CircRNAs as diagnostic biomarkers for tuberculosis

Some circRNAs are known to be expressed in a disease-specific manner. Combined with other features of circRNAs including conservation, stability and high abundance in body fluids, circRNAs are believed to be promising biomarkers for various diseases including TB (61) (**Table 1**). Using whole transcriptome sequencing, Zhang et al. identified 170 dysregulated circRNAs in whole blood samples from pulmonary TB patients, compared with samples in health individuals (69). Their findings suggested that circRNA-linked competing endogenous RNAs (ceRNA) -mediated regulation of gene is critical for pulmonary TB pathogenesis. Using microarray and quantitative real-time PCR analysis, Huang et al. revealed that a number of circRNAs were differentially expressed in PBMCs from active TB patients. Among them, hsa_ circ_001937 exhibits a substantial diagnostic value for TB with an area-under-curve (AUC) value of 0.873 in the receiver operating characteristic (ROC) curve analysis (63). It was specifically upregulated in patients with TB compared with patients with other lung diseases, and was also correlated with TB severity (63). Yi et al. found that the level of hsa_circRNA_103571 significantly decreased in active TB plasma samples and could be served as a potential biomarker for active TB diagnosis with an AUC value of 0.838 (47). Zhuang et al. reported that hsa_circ_0009128 and hsa_circ_0005836 were significantly down-regulated in the PBMCs of active pulmonary TB (APTB) patients compared with health controls and the hsa_circ_0005836 might act as novel potential biomarkers for APTB (64). Recently, this group reported hsa_circ_0001380 was also significantly downregulated in the PBMCs from TB patients compared with healthy individuals. The ROC curve analysis revealed that the AUC value for distinguishing APTB using hsa_circ_0001380 was 0.9502, indicating the high diagnostic value of this circRNA in APTB (65). Another study identified three differentially expressed cicrRNAs by analyzing public GEO datasets and found hsa_circ_0028883 showed a potential diagnostic value in active TB with an AUC value of 0.773 (66). Additionally, Fu et al. characterized the expression profiles of circRNAs in PBMCs of active TB patients and discovered 171 circRNAs were dysregulated in TB samples. Among them, circRNA_059914, circRNA_101128 and circRNA_103017 were significantly upregulated and showed a potential diagnostic power with an AUC value of 0.87, 0.821 and 0.817 respectively (48).

**Table 1:** CircRNAs as tuberculosis diagnostic biomarker.

CircRNAs	Dysregulation	Samples	Detection method	Patients (n)	Diagnostic value	Ref.
hsa_circ_0043497	up	PBMC	qRT-PCR	96	AUC 0.86	(62)
hsa_circ_0001204	down	PBMC	qRT-PCR	96	AUC 0.848	(62)
hsa_circRNA_001937	up	PBMC	qRT-PCR	115	AUC 0.873; sensitivity 72.2%, specificity 90%	(63)
hsa_circRNA_103571	down	plasma	qRT‐PCR	32	AUC 0.838	(47)
Hsa_circ_0005836	down	PBMC	RNAseq,qRT-PCR	49	NA	(64)
hsa_circ_0001380	down	PBMC	qRT‐PCR	32	AUC 0.9502, sensitivity 93.75%, specificity 87.5%	(65)
hsa_circ_0028883	up	PBMC	qRT‐PCR	20	AUC 0.773	(66)
circRNA_103017	up	PBMC	qRT‐PCR	31	AUC 0.87	(48)
circRNA_059914	up	PBMC	qRT‐PCR	31	AUC 0.821	(48)
circRNA_101128	up	PBMC	qRT‐PCR	31	AUC 0.817	(48)
hsa_circ_0001204	down	plasma	qRT‐PCR	145	AUC 0.871 sensitivity 73.1%, specificity 92.5%	(67)
hsa_circ_0001747	down	plasma	qRT‐PCR	145	AUC 0.83 sensitivity 71.03%, specificity 82.5%	(67)
hsa_circ_0001204, hsa_circ_0001747	down	plasma	qRT‐PCR	145	AUC0.928, sensitivity 86.21%, specificity 89.17%	(67)
7-circRNA signature	up	PBMC	qRT‐PCR	10	AUC 0.946,	(68)
circRNA_051239	up	serum	qRT‐PCR	128	AUC 0.9738	(55)
circRNA_029965	up	serum	qRT‐PCR	128	AUC 0.9443	(55)
circRNA_404022	up	serum	qRT‐PCR	128	AUC 0.9682	(55)
circRNA_051239, circRNA_029965, circRNA_404022	up	serum	qRT‐PCR	128	AUC 0.9920	(55)

In addition to individual circRNA, the combination of circRNAs has shown a better predictive power in TB diagnosis. One study reported the expression of two circRNAs (hsa_circ_0001204 and hsa_circ_0001747) was significantly decreased in active TB plasma samples compared with health controls. The ROC curve analysis exhibited an AUC value of 0.928 for distinguishing TB patients when hsa_circ_0001747 and hsa_circ_0001204 were used in combination, indicating this circRNA combination could act as a novel biomarker for active TB diagnosis (67). Qian et al. also identified differentially expressed circRNAs in pulmonary TB patients’ PMBCs. Among them, seven circRNAs including hsa_circ_0000414, hsa_circ_0002908, hsa_circ_0000681, hsa_circ_0002362, hsa_circ_0002113, hsa_circ_0008797 and hsa_circ_0063179 were chosen to develop a circRNA-based molecular signature based on pathway analysis. In validated groups, the 7-circRNA-based TB index was significantly higher in TB patients than that in healthy controls with an AUC value of 0.946 (68). Furthermore, Liu *et al.* identified three circRNAs (circRNA_029965, circRNA_051239 and circRNA_404022) which showed significantly increases in the serum of the active TB patients. The AUC value of the combination of these three circRNAs in ROC curve analysis is as high as 0.992, suggesting these cicrRNAs could serve as ideal potential biomarkers for TB diagnosis (55).

## Discussion

The current advanced RNA-sequencing technologies and data analysis algorithms have largely promoted the discovery of host circRNAs. These circRNAs, at least some of them, are now considered to have important functions in the process of Mtb infection. However, current studies on the roles of circRNAs in TB are mainly focused on autophagy and macrophage polarization. Exact mechanisms of circRNAs’ action are still largely unknown. The roles of circRNAs in other immune processes during Mtb infection and their mechanisms need to be comprehensively studied. Also, new technologies, analyses and strategies are still needed to identify key functional circRNAs in TB.

Due to their excellent high abundance and stability in body fluids, circRNAs are regarded as promising diagnostic biomarkers for human diseases including TB. Indeed, as listed in **Table 1**, dozens of circRNAs or combinations are reported as biomarkers for the diagnosis of TB with varied predictive power. However, several problems/challenges still need to be addressed in the future. Firstly, the samples sizes of current published studies are relatively small. Muti-center large cohort studies are needed to comprehensively evaluate the diagnostic value of these circRNAs. The sensitivity and reliability of using circRNAs as diagnostic biomarkers need further validation. Secondly, as the absolute expression level of circRNAs in samples may vary from person to person, it may be difficult to set up a normal baseline for distinguishing patients from healthy people. Thus, a standardized protocol for circRNAs detection in clinical samples is required. Finally, detection of circRNAs in clinical samples is more expensive and time-consuming currently than protein detection, which may limit the application of circRNAs as biomarkers. It is very important to be considered as the fact that over 95% TB patients are in developing countries.

In addition to being diagnostic biomarkers, circRNAs could be as potential therapeutic targets or therapeutic strategies for tuberculosis. The disease-promoting circRNAs could be knocked down by RNAi or CRISPR/Cas9-based gene editing. The therapeutic circRNAs could be designed and synthesized artificially according to the clinical need. Liu et al. artificially synthesized a circRNA for the first time and showed this circRNA could inhibit the proliferation of gastric cancer cells *in vitro* by sponging miR-21 (70). Furthermore, a very recent elegant study found the circRNA vaccine against SARS-CoV-2 enabled higher and more durable antigen production than the modified mRNA vaccine and induced a higher neutralizing antibody titers (71). This study provided a novel idea for the potential application of circRNAs in developing TB vaccine in the future.

## Author contributions

QW, DY, YZ, and DW drafted the manuscript. QW and WL supervised and edited the manuscript. All authors contributed to the article and approved the submitted version.

## Funding

This work was supported by the startup fund from West China Hospital to Qinglan Wang (137210102).

## Conflict of interest

The authors declare that the research was conducted in the absence of any commercial or financial relationships that could be construed as a potential conflict of interest.

## Publisher’s note

All claims expressed in this article are solely those of the authors and do not necessarily represent those of their affiliated organizations, or those of the publisher, the editors and the reviewers. Any product that may be evaluated in this article, or claim that may be made by its manufacturer, is not guaranteed or endorsed by the publisher.
